# Supplementary material for: Fear of pain moderates the relationship between self-reported fatigue and methionine allele of catechol-O-methyltransferase gene in patients with fibromyalgia
Source: PLoS One. 2021 Apr 28;16(4):e0250547. doi: 10.1371/journal.pone.0250547 (PMC8081450; doi:10.1371/journal.pone.0250547)
Supplement: S1 Table — P-values of main effects of the COMT groups are also included. (DOCX) [file pone.0250547.s001.docx]

**S1 Table. Means and standard deviation (in parenthesis) of clinical measures of COMT groups. P-values of main effects of COMT group are also included.**

|  | Met/Met | Met/Val | Val/Val | p-value |
| --- | --- | --- | --- | --- |
| STAI State | 42.09 (28.40) | 39.85 (29.02) | 44.29 (27.61) | 0.786 |
| STAI Trait | 57.39 (30.71) | 49.33 (33.68) | 55.61 (33.41) | 0.384 |
| BDI | 18.60 (15.71) | 16.87 (14.84) | 20.88 (18.49) | 0.683 |
| PCS Total | 47.63 (26.84) | 39.56 (29.08) | 43.72 (30.01) | 0.395 |
| PCS Rumiation | 41.81 (27.11) | 38.97 (29.38) | 40.40 (29.57) | 0.903 |
| PCS Magnification | 53.23 (24.62) | 53.75 (27.90) | 44.42 (27.59) | 0.244 |
| PCS Helplessness | 53.26 (28.96) | 41.63 (28.15) | 46.37 (29.73) | 0.132 |
| FPQ-III Total | 78.81 (22.41) | 70.74 (21.56) | 75.88 (19.10) | 0.256 |
| FPQ-III Severe | 32.37 (10.25) | 30.84 (10.77) | 32.69 (8.65) | 0.825 |
| FPQ-III Minor | 21.81 (8.23) | 18.63 (6.74) | 19.97 (6.19) | 0.090 |
| FPQ-III Medical | 24.54 (7.45) | 21.22 (6.63) | 23.22 (6.86) | 0.061 |
| TSK | 37.31 (8.77) | 36.40 (11.50) | 35.54 (12.87) | 0.107 |
| VAS Pain | 4.30 (3.46) | 3.48 (3.22) | 4.47 (3.65) | 0.152 |
| VAS Fatigue | 3.94 (3.56) | 3.05 (2.79) | 4.21 (3.38) | 0.167 |
