# Supplementary material for: Fear of pain moderates the relationship between self-reported fatigue and methionine allele of catechol-O-methyltransferase gene in patients with fibromyalgia
Source: PLoS One. 2021 Apr 28;16(4):e0250547. doi: 10.1371/journal.pone.0250547 (PMC8081450; doi:10.1371/journal.pone.0250547)
Supplement: Attachment — Submitted filename: Response to Reviewers.docx [file pone.0250547.s003.docx]

**Author's response to reviewers**

ID PONE-D-20-07498R1

"Fear of pain moderates the relationship between self-reported fatigue and methionine allele of catechol-O-methyltransferase gene in patients with fibromyalgia"

**Authors:**

David Ferrera ([david.ferrera@urjc.es](mailto:david.ferrera@urjc.es))

Francisco Mercado

Irene Peláez

David Martínez-Íñigo

Roberto Fernandes-Magalhaes

Paloma Barjola

Carmen Écija

Gema Díaz-Gil

Francisco Gómez-Esquer

**Version**: 3 **Date**: February 1, 2021

Dr. Mahmoud Abu-Shakra, MD

Academic Editor

PLOS ONE

Dear Dr. Abu-Shakra,

Thank you very much for your e-mail and comments submitted by the reviewer #2. We have responded to the reviewer's comments and suggestions point by point and we hope that this new version is acceptable for publication in PLOS ONE. We would like to thank you again for your consideration of our research and inviting us to submit the revised manuscript.

Best regards,

David Ferrera

----------------------------------------------------------------------------------------------------------

- **Reviewer #2**

**Point 16 - The main conclusion is now phrased a bit more careful, but still confusing: “Main results indicated that patients carrying the Met/Met genotype reported significantly higher levels of fatigue compared to heterozygotes carriers and higher, but not significant, scores than Val homozygotes carriers.” I think this should be: “and higher, but not significantly different from Val homozygote carriers” – or something along this line. Note that addressing my next point could actually also be relevant for this point (if it turns out that Val/Val carriers do show higher fatigue and pain compared to the other groups combined).**

Response: Thank you for the suggestion. Following this recommendation, we rephrased the Abstract (see page 2, lines 39) and the Conclusion (page 23-24, line 513-514) sections in order to make them more understandable.

**Point 17 – I appreciate the explanation and do understand the rationale for taking Met/Met and Val/Met together (representing the Val158Met polymorphism carriers if I understand it correctly now). However, the use of two different approaches in this manuscript is still problematic if this is not explicitly explained nor addressed.**

**If the authors want to keep both strategies (use 3 genotype groups for part A-group differences, and use 2 genotype groups for part B-moderation), it should be a) explicitly acknowledged that two different groupings are used (3 groups versus 2 groups) and why based on provided references e.g., and b) the 2 genotype groups should be formally (statistically) compared to see if results hold up when using this different grouping (i.e., to see if Val/Val carriers indeed show higher self-reported pain and fatigue than the other group) in order to justify using this alternative grouping for part B-moderation.**

**The current strategy is problematic as the moderation analyses build on the group difference findings, but in fact, they cannot be built on this due to different analytical strategies. And the analyses will be invalid.**

**The alternative would be that the analyses that are described in the response letter are presented in the paper as a way to justify using the different groupings.**

Response: Thank you for your comments. We agree with the reviewer that the use of both strategies might be confusing. However, this approach combining the use of two analysis strategies has been regularly used to explore the relationship between genetic factors and clinical/cognitive variables in pain. Some articles where this type of analysis has been carried out are the following:

-Cargnin, S., Magnani, F., Viana, M., Tassorelli, C., Mittino, D., Cantello, R., Sances, G., Nappi, G., Canonico, P. L., Genazzani, A. A., Raffaeli, W., & Terrazzino, S. (2013). An Opposite-Direction Modulation of the COMT Val158Met Polymorphism on the Clinical Response to Intrathecal Morphine and Triptans. *The Journal of Pain*, *14*(10), 1097–1106. https://doi.org/10.1016/j.jpain.2013.04.006

-Cohen, H., Neumann, L., Glazer, Y., Ebstein, R. P., & Buskila, D. (2009). The relationship between a common catechol-O-methyltransferase (COMT) polymorphism val158met and fibromyalgia. *Clinical and Experimental Rheumatology*, *27*(5), S51–S56.

-Gürsoy, S., Erdal, E., Herken, H., Madenci, E., Alaşehirli, B., & Erdal, N. (2003). Significance of catechol-O-methyltransferase gene polymorphism in fibromyalgia syndrome. *Rheumatology International*, *23*(3), 104–107. https://doi.org/10.1007/s00296-002-0260-5

-Lee, Y. H., Kim, J.-H., & Song, G. G. (2015). Association between the COMT Val158Met polymorphism and fibromyalgia susceptibility and fibromyalgia impact questionnaire score: a meta-analysis. *Rheumatology International*, *35*(1), 159–166. https://doi.org/10.1007/s00296-014-3075-2

-Sindermann, C., Luo, R., Zhang, Y., Kendrick, K. M., Becker, B., & Montag, C. (2018). The COMT Val158Met Polymorphism and Reaction to a Transgression: Findings of Genetic Associations in Both Chinese and German Samples. *Frontiers in Behavioral Neuroscience*, *12*. https://doi.org/10.3389/fnbeh.2018.00148

Although we have decided to keep the original strategy, several changes have been introduced in different parts of the manuscript, as recommended by the reviewer's suggestions. Specifically, we have added a theoretical explanation to justify why this approach might be a valid grouping strategy, being supported by a well-established physiological explanation (see pages 3-4, lines 72-80). On the other hand, we conducted a new moderation analysis (Results section). to explore the role of the psychological symptoms as potential factors to moderate the relationship between pain/fatigue and the three COMT genotypes (i.e., Met/Met, Met/Val and Val/Val) within the sample of patients with fibromyalgia (see pages 15, lines 324-329). The results derived from these analyses did not show any significant differences. Thus, we kept the grouping strategy including methionine carriers into a single group (Met/Met, Met/Val) and Valine homozygotes (Va/Val) in a different one. Moreover, the use of this strategy has recently been recommended in fibromyalgia. Lee and colleagues (2015) explored the associations between the Val158Met polymorphism and the risk for suffering fibromyalgia. A total of 1531 fibromyalgia patients from 15 studies were included in this meta-analysis. It revealed an association between fibromyalgia and the Met/Met + Val/Met genotypes in the whole sample of patients. The Met allele of the COMT gene was associated with a higher impact of fibromyalgia compared to Val allele. Considering all this information, we have proceeded to edit the Discussion section trying to integrate our results with previous evidence providing similar findings (pages 21-22, lines 457-470).

**Point 18 - The authors have changed wording to ‘patients/individuals with fibromyalgia’ and avoided ‘subjects’ for the most part. Yet, I would recommend them to do another check as some old terms are remaining (e.g., line 103, 354 or 421) and also the title still uses the old style. Also, talking about the title: I am not sure ‘fatigue perception’ is an appropriate term in this case.**

**The same with ‘catastrophism’ which is still used in the manuscript.**

Response: Thank you for the reviewer’s suggestion. We have removed the term *subjects* in the entire manuscript. Likewise, we have changed the title and we have replaced the term *fibromyalgia subjects* for the term *patients with fibromyalgia*. In addition, also in the title, we have replaced the concept of *fatigue perception* by the term *self-reported fatigue*, as it has been used in the rest of the text. Finally, we have edited the manuscript to unify the use of the term *catastrophism*. We have used the term *pain catastrophizing* in the entire manuscript to refer this variable.

**Tables - The authors did a good job updating the result sections and corresponding tables.**

**Point 26 – It is still unclear how the Hardy-Weinberg equilibrium was tested. What was the expected distribution? And also, some of this discussion in the response letter would merit inclusion in the manuscript.**

Response: We have included additional information on the purpose of the Hardy-Weinberg analysis in the Method section (pages 8-9, lines 195-202). In brief, Hardy-Weinberg method is a control analysis that is frequently conducted to analyse the distribution of genotypes within a given sample. Investigations focused on the study of associations between the presence of a disease and different genetic factors should met this equilibrium for the sample of healthy participants. It can also be done in the patients’ sample in order to explore if a specific gene could be associated with the development of a given disease. It would be an interesting question to explore in further investigations. In the current study, our aim was rather to explore the relationship between COMT gene and different symptoms characterizing this disease. For this reason, although following the reviewer’s suggestions we have added additional information about the purpose of this control analysis, we have not discussed about the distribution of genotypes in fibromyalgia since it adjusts to Hardy-Weinberg equilibrium.

**Point 37 – Wonderful. This would make an excellent contribution to the manuscript, so I would suggest including it.**

Response: Following the reviewer's recommendations we have edited the manuscript including this information. We have added a paragraph in the Method section (see pages 9, lines 211-216), as well as the data corresponding to the correlation analysis (see Results section; page 14, lines 299-308).

**Point 41 – This is a nice discussion that also deserves to be added to the manuscript. Because no matter how you look at it, the one (and only) variable that is not elevated in the patients compared to controls does act as a moderator variable (even though analysis strategies are different). Discussion on why this would be, or at least acknowledging this, is very relevant.**

Response: Following the recommendations given by the reviewer, we have added in the Discussion section, this information. This new information can be consulted on pages 22, lines 471-482.

**General point - One of my struggles was with the use of terms such as genotype, (Methionine) alleles, polymorphisms, heterozygotes carriers, Val158Met, etc of which some seem to be synonyms, but others mean slightly different things.**

**Many things have been clarified in the responses as well as in the manuscript, for which I am grateful, but it would greatly improve the comprehensiveness of the manuscript if there was some consistency in referring to some of these jargon terms, especially in a journal like this which is read by a broad audience.**

Response: Thanks for the reviewer's suggestion. We have revised the entire manuscript in order to clarify some of these terms. As the reviewer points out, PLOS ONE is a journal with a wide audience, but that fact should not undermine the use of appropriate terms, even if they are technical, to maintain certain of scientific quality. For that reason, we have maintained some these terms in the manuscript.

Among other things, we have included the word polymorphism after the term Val158Met (e.g., see page 3, line 62 or page 5, line 97). Also, we have clarified that the Met alleles involve both the carriers of the Met/Met and Met/Val genotypes (e.g., page 3, line 68 or page 22, line 466). Finally, we have added the term gene after COMT in different parts of the manuscript (e.g., page 5 line 110 and 111).

**Minor points**

***Abstract line 33 “influences of COMT genotypes and group of participants” – does the latter refer to comparing patients with controls? Please rephrase.**

Response: Thank you for your helpful comment. We have changed this sentence to reflect that the analysis shows the comparison between healthy participants and patients with fibromyalgia (see page 2, line 33-34).

***Page 6, lines 138-142 – This is not the place for this information, this should rather be placed at the description of the sample (around Table 2 e.g.)**

Response: Thanks for your suggestion. As the reviewer indicates, we have moved the age analysis to the results section (page 11, line 251-254).

***Page 12, lines 265-266 – Please include the comparison Met/Met and Val/Val**

Response: Following the reviewer's recommendations, we have included the comparison between homozygous subjects on page 13, lines 283-284.

***Page 12, line 268 – Please avoid qualifying words such as a ‘strong’ trend.**

Response: Thank you for your comment. We have revised the manuscript and we have removed these qualifying words.

***Page 12 – It is unclear whether there were main effects of COMT gene (across groups).**

Response: As you can see on the page 13, lines 290-291, there is information about the main effects of COMT genotype.
