# Supplementary material for: Fear of pain moderates the relationship between self-reported fatigue and methionine allele of catechol-O-methyltransferase gene in patients with fibromyalgia
Source: PLoS One. 2021 Apr 28;16(4):e0250547. doi: 10.1371/journal.pone.0250547 (PMC8081450; doi:10.1371/journal.pone.0250547)
Supplement: Attachment — Submitted filename: Response to Reviewers.docx [file pone.0250547.s004.docx]

**Author's response to reviewers**

ID PONE-D-20-07498R2

"Fear of pain moderates the relationship between self-reported fatigue and methionine allele of catechol-O-methyltransferase gene in patients with fibromyalgia"

**Authors:**

David Ferrera ([david.ferrera@urjc.es](mailto:david.ferrera@urjc.es))

Francisco Mercado

Irene Peláez

David Martínez-Íñigo

Roberto Fernandes-Magalhaes

Paloma Barjola

Carmen Écija

Gema Díaz-Gil

Francisco Gómez-Esquer

**Version**: 4 **Date**: April 1, 2021

Dr. Mahmoud Abu-Shakra, MD

Academic Editor

PLOS ONE

Dear Dr. Abu-Shakra,

Thank you very much for your e-mail and comments submitted by the reviewers. Following your and the reviewer #2 suggestions, we have proceeded to carry out a professional English proofreading of the entire manuscript. We believe that our manuscript has been considerably improved as a result of these revisions and hope that this new version is acceptable for publication in PLOS ONE. We would like to thank you again for your consideration of our research and inviting us to submit the revised manuscript.

Best regards,

David Ferrera
